# Isaridin E Protects Against UVB-Induced Photoaging by Activating Wnt/β-Catenin Signaling Pathway and Alleviating Mitochondrial Dysfunction

**DOI:** 10.3390/md24030112

**Published:** 2026-03-18

**Authors:** Yaosheng Liu, Weizhen Li, Zeen Yang, Hui Long, Sufen Cai, Changjie Sun, Yu Xiong, Yunqi Zhang, Yumei Liu, Guangpu Luo, Senhua Chen, Tie Zhao

**Affiliations:** 1 Institute of Dermatology, Guangzhou Medical University, Guangzhou 510095, China; liuysh55@gzhmu.edu.cn (Y.L.); 2024211760@stu.gzhmu.edu.cn (W.L.); yangzeen@gzhmu.edu.cn (Z.Y.); 2017690096@gzhmu.edu.cn (H.L.); 2017690098@gzhmu.edu.cn (S.C.); sunchj@gzhmu.edu.cn (C.S.); 2024622436@gzhmu.edu.cn (Y.X.); 2023621198@gzhmu.edu.cn (Y.Z.); 2014690016@gzhmu.edu.cn (Y.L.); 2025622038@gzhmu.edu.cn (G.L.); 2 Department of Pharmacology, Zhongshan School of Medicine, Sun Yat-sen University, Guangzhou 510080, China; 3 School of Pharmaceutical Sciences, Guangzhou Medical University, Guangzhou 511436, China; 4 School of Marine Sciences, Sun Yat-Sen University, Zhuhai 519082, China; 5 Southern Marine Sciences and Engineering Guangdong Laboratory (Zhuhai), Zhuhai 519000, China

**Keywords:** photoaging, mitochondrial dysfunction, Wnt/β-catenin, ultraviolet B, Isaridin E

## Abstract

Mitochondrial dysfunction is a major contributor to skin photoaging. Activation of the Wnt/β-catenin pathway, a key regulator of developmental processes, can improve mitochondrial abnormalities associated with pathology. Therefore, the Wnt/β-catenin pathway emerges as a key therapeutic target in the context of photoaging. Isaridin E (ISE), a marine-derived natural product with a novel structure, exhibits potent antiplatelet and anti-inflammatory activities. We sought to examine the anti-senescence effects of ISE on fibroblasts in photoaged skin. In vitro, ISE improved UVB-induced fibroblast damage in a dose-dependent manner, restoring cell viability, reducing β-galactosidase accumulation, and suppressing SASP factor production. In a photoaging mouse model, ISE markedly decreased skin thickness, increased dermal collagen expression, and reduced SASP levels in skin tissues. ISE significantly improved fibroblast energy production deficits and mitochondrial dysfunction. RNA sequencing and Western blotting demonstrated that UVB irradiation significantly suppressed Wnt/β-catenin signaling activity, whereas ISE dose-dependently restored pathway activation. Using GSK-3β-targeted siRNA, we showed that the anti-photoaging effects of ISE are mediated via the Wnt/β-catenin pathway. ISE appears to counteract photoaging by enhancing Wnt/β-catenin activity and improving mitochondrial function.

## 1. Introduction

Photoaging is characterized by progressive alterations in skin structure and function induced by prolonged solar irradiation. These alterations include skin wrinkles and impaired structural barrier [1]. Moreover, photoaging is strongly associated with an increased risk of skin malignancies, including basal cell carcinoma and melanoma [1]. Despite its high prevalence and substantial potential risks, effective therapeutic options remain limited, underscoring an urgency to identify novel therapeutic targets and intervention strategies.

Fibroblasts, which dominate the dermal cellular landscape, generate extensive collagen networks and additional matrix elements critical for preserving skin architecture and homeostatic regulation [2]. Long-term UVB exposure causes mitochondrial dysfunction in dermal fibroblasts, leading to reactive oxygen species (ROS) accumulation and depletion of endogenous antioxidants such as superoxide dismutase (SOD) and glutathione (GSH), thereby triggering oxidative stress [1,3]. This consequent redox imbalance induces damage to nuclear and mitochondrial DNA (mtDNA), initiating the p53-p21 signaling cascade. The resultant suppression of cyclin-dependent kinases imposes cell-cycle arrest, suppressing collagen synthesis [4,5,6]. In addition, oxidative stress promotes the formation of the SASP [7]. SASP factors, including pro-inflammatory cytokines (such as interleukin (IL)-6, IL-1β, and tumor necrosis factor-alpha (TNF-α)) and matrix-degrading enzymes (such as MMP3 and MMP9), exacerbate collagen degradation and extracellular matrix destruction [8]. In addition, SASP-driven inflammatory signaling further increases ROS production, creating a positive feedback loop that worsens dermal matrix breakdown and promotes skin aging [9]. Therapeutic modulation of mitochondrial dysfunction may ease photoaging via attenuation of DNA damage and restraint of SASP-associated secretory activity [9,10]. An enhanced understanding of the molecular mechanisms underlying mitochondrial dysfunction may reveal new therapeutic targets for photoaging.

Wnt/β-catenin signaling is essential for tissue homeostasis, as it controls key processes such as cell proliferation, differentiation, and stem cell maintenance [11]. In the skin, Wnt/β-catenin signaling sustains fibroblast activity, extracellular matrix production, and wound healing, whereas its dysregulation contributes to impaired regeneration, photoaging and the development of various cancers [11,12]. GSK-3β is a core negative modulator in the canonical Wnt/β-catenin pathway [11]. GSK-3β phosphorylates β-catenin, promoting its degradation and lowering intracellular β-catenin levels. Upon Wnt/β-catenin activation, this phosphorylation is suppressed, allowing β-catenin to accumulate and translocate into the nucleus to drive TCF/LEF-mediated transcription [13]. Thus, modulating Wnt/β-catenin signaling may offer therapeutic benefits in a range of dermatological conditions, such as hypertrophic scars and skin malignancies [12]. Additionally, Oxidative stress modulates Wnt/β-catenin signaling through redox-sensitive mechanisms [14]. However, the pathological significance of the Wnt/β-catenin pathway in skin photoaging and the underlying molecular mechanism remain unclear.

ISE, a cyclodepsipeptide of fungal origin, was characterized as the principal secondary metabolite of *Amphichorda felina* (*syn. Beauveria felina*) SYSU-MS7908, a marine-derived fungus isolated from Styela plicata inhabiting the North Atoll of the Xisha Islands, South China Sea (17°06′14.50″ N, 111°28′35.03″ E) [15]. Our previous studies demonstrated that ISE effectively inhibits arterial thrombosis without prolonging bleeding time, indicating its promising therapeutic potential [16]. Moreover, in a CLP-induced arterial thrombosis model, ISE significantly attenuated sepsis-associated inflammatory responses [17]. Fang-Rong Chang et al. reported that ISE exhibits anti-inflammatory effects by suppressing oxidative stress without detectable cytotoxicity [18]. Oxidative stress accelerates the progression of skin photoaging, which is closely related to fibroblast SASP. In turn, SASP stimulates excessive ROS production, amplifying oxidative stress. Given its potent inhibitory effect on oxidative stress, we hypothesized that ISE attenuates fibroblast SASP and collagen degeneration in skin photoaging.

In this study, we aimed to explore the efficacy of ISE against photoaging and elucidate the relevant underlying molecular mechanisms.

## 2. Results

### 2.1. ISE Effectively Protected Fibroblasts Against UVB-Induced Photoaging In Vitro

**Figure 1:**
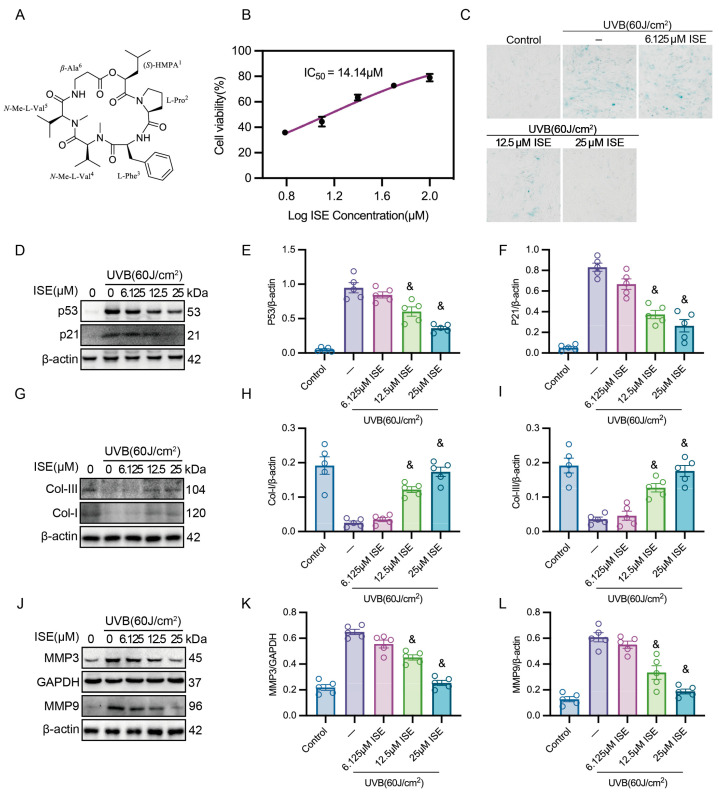
ISE significantly improved UVB-induced photoaging. (**A**) Chemical structural formula of ISE. (**B**) Cellular metabolic activity was measured using the 3-(4,5-dimethylthiazol-2-yl)-2,5-diphenyltetrazolium bromide assay. (**C**) Representative images of SA-*β*-galactosidase staining of MDFs in each group. (**D**–**F**) Western blotting representative images and bar charts showing the total P21 and P53 proteins of MDFs in each group. (**G**–**I**) Western blotting of Collagen I and III expression in MDFs in each group. (**J**–**L**) Representative Western blotting and quantitative bar graphs showing MMP3 and MMP9 total proteins in MDFs among different groups. Data are presented as mean ± SEM. *n* = 5 independent experiments. & denotes *p* < 0.05 vs. the UVB group.

ISE, a colorless crystalline compound with the molecular formula C_35_H_54_O_7_N_5_, was utilized in this study. The chemical structure of ISE is depicted in Figure 1A. To evaluate the effects of ISE on fibroblasts after UVB irradiation, we first used the MTT assay to assess cellular metabolic activity as an indicator of cell viability. As shown in Figure 1B, ISE markedly alleviated the UVB-induced reduction in cellular metabolic activity in a concentration-dependent manner. As the concentration of ISE increased, the MTT signal gradually recovered, and the protective IC_50_ was calculated to be 14.14 µM. Subsequently, β-galactosidase staining demonstrated the potential of ISE to mitigate UVB-induced cellular senescence. The β-galactosidase-positive area progressively decreased with increasing ISE concentrations, indicating that ISE effectively attenuated UVB-induced senescence (Figure 1C). In addition, Western blotting showed the protein expression of P53 and P21, key regulators of the G1/S cell-cycle checkpoint and well-established markers of cellular senescence. UVB irradiation markedly elevated P53 and P21 expression in fibroblasts, whereas ISE dose-dependently suppressed their expression (Figure 1D–F). Collagen I and III synthesis is a major function of fibroblasts and is essential for maintaining skin structure and function. Western blotting revealed that UVB exposure decreased the synthesis of Collagen I and III, while ISE significantly restored their production in a dose-dependent manner (Figure 1G–I). MMP3 and MMP9 are representative SASP molecules. ISE significantly reduced the UVB-induced elevation of MMP3 and MMP9 protein levels (Figure 1J–L). These findings demonstrate that ISE markedly ameliorates UVB-induced photoaging in fibroblasts in vitro.

### 2.2. ISE Effectively Protected Fibroblasts Against UVB-Induced Mitochondrial Dysfunction In Vitro

**Figure 2:**
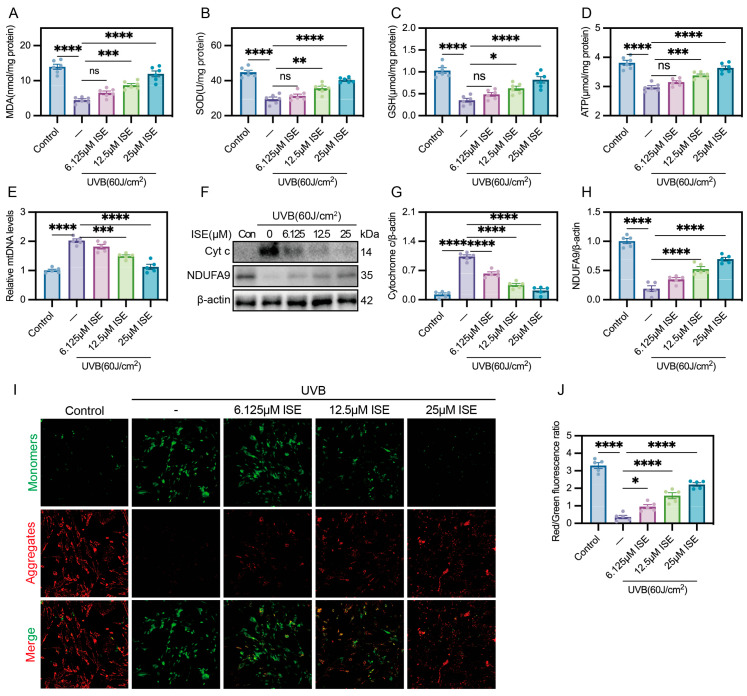
Assessment of ISE in preventing UVB-induced mitochondrial dysfunction in vitro. (**A**–**E**) Bar charts showing malondialdehyde, superoxide dismutase, glutathione, ATP, and mtDNA levels in MDFs in each group. (**F**–**H**) Representative Western blotting and quantitative bar graphs showing Cytochrome c and NDUFA9 total protein in MDFs among different groups. (**I**,**J**) Representative images of JC-1 staining and quantification of the red/green fluorescence intensity ratio. Scale bars = 50 µm. Aggregates, red; monomers, green. Data are presented as mean ± SEM. *n* = 5 independent experiments. ns represent *p >* 0.05, * *p <* 0.05, ** *p <* 0.01, *** *p <* 0.001, **** *p <* 0.0001.

Mitochondrial dysfunction is a key mechanism underlying photoaging. UVB irradiation caused a marked decrease in intracellular antioxidants, including malondialdehyde (MDA), SOD, and GSH, accompanied by reduced ATP generation, which were ameliorated by ISE in a dose-dependently manner (Figure 2A–D). Quantitative polymerase chain reaction on the mtDNA levels showed that treatment with 12.5 and 25 µM ISE reduced the mtDNA levels in the UVB group (2.02 ± 0.14) to 1.49 ± 0.10 and 1.13 ± 0.19, respectively (Figure 2E). After UVB irradiation, NDUFA9 was downregulated, accompanied by an elevated Cytochrome c expression; these events were ameliorated by ISE (Figure 2F–H). In addition, JC-1 staining demonstrated that ISE modulated UVB-induced alterations in mitochondrial membrane potential. As shown in Figure 2I,J, JC-1 aggregates are indicated by red fluorescence, while monomeric JC-1 emits green fluorescence. UVB irradiation markedly reduced the JC-1 aggregate/monomer fluorescence ratio, while increasing concentrations of ISE progressively restored this ratio. These results demonstrate that ISE effectively protects fibroblasts from UVB-induced mitochondrial dysfunction in vitro.

### 2.3. Wnt/β-Catenin Signaling Pathway Was Restored by ISE in Photoaged Fibroblasts

**Figure 3:**
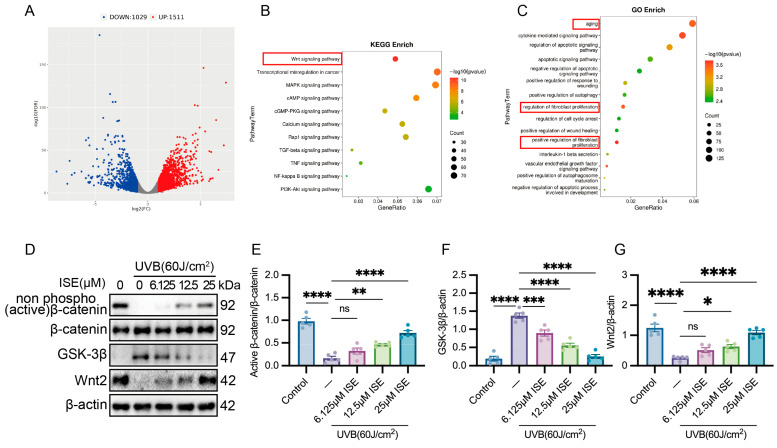
ISE significantly alleviated the suppression of the Wnt/β-catenin signaling pathway in UVB-induced MDFs. (**A**) Volcano plot of the RNA sequencing of vehicle- or ISE-treated MDFs exposed to UVB. (**B**,**C**) Kyoto Encyclopedia of Genes and Genomes and Gene Ontology enrichment analyses revealed that ISE regulated various biological processes in UVB-induced MDFs. *n* = 3 independent experiments (**D**–**G**). Representative Western blotting and quantitative bar graphs showing Wnt/β-catenin signaling in MDFs between different groups. Data are presented as mean ± SEM. *n* = 5 independent experiments. ns: not significant, * *p <* 0.05, ** *p <* 0.01, *** *p <* 0.001, **** *p <* 0.0001.

Transcriptomic analysis of the ISE-treated and untreated UVB groups revealed the mechanisms by which ISE influences photoaging. Differential gene expression analysis identified 2540 genes, of which 1511 were upregulated and 1029 were downregulated. (Figure 3A). KEGG pathway analysis revealed that the DEGs were mainly enriched in several pathways, with the Wnt signaling pathway being one of the most significant (Figure 3B). Gene Ontology (GO) enrichment analysis indicated that these DEGs were closely related to various biological processes, including aging and the regulation of fibroblast proliferation (Figure 3C). Based on the established contribution of Wnt signaling to aging [19], Wnt pathway-associated differentially expressed genes were analyzed to uncover putative targets of ISE.

Western blot analysis revealed that UVB exposure significantly reduced actived β-catenin and Wnt2 protein levels and upregulated GSK-3β. (Figure 3D–G). Increasing ISE concentrations suppressed the GSK-3β level, while the protein levels of activated β-catenin and Wnt2 progressively increased.

### 2.4. Anti-Photoaging Effect of ISE Depended on the Activation of Wnt/β-Catenin Signaling Pathway

**Figure 4:**
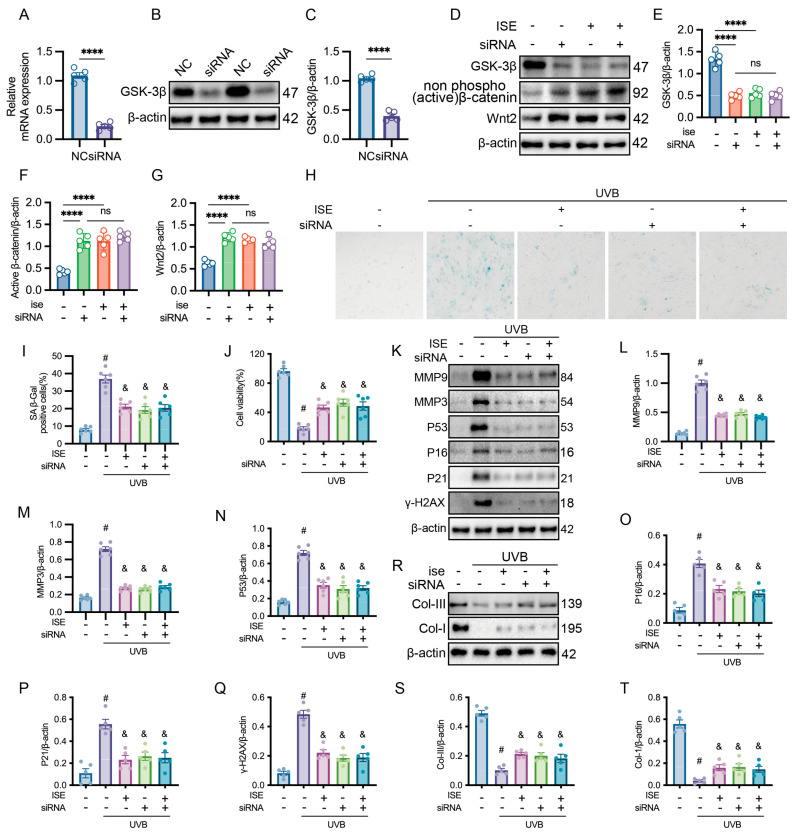
Effect of siRNA targeting β-catenin on the anti-photoaging ability of MDFs in vitro. (**A**) Relative mRNA expression of GSK-3β in MDFs transfected with negative control (NC) or GSK-3β-specific siRNA, as determined by qRT-PCR. (**B**,**C**) Representative Western blot images showing GSK-3β protein levels following NC or siRNA transfection, with β-actin as a loading control. (**D**–**G**) Representative Western blot analysis of GSK-3β, non-phosphorylated (active) β-catenin, and Wnt2 under the indicated conditions with or without ISE treatment and GSK-3β siRNA transfection. *n* = 5 independent experiments. ns: not significant, **** *p <* 0.0001. (**H**,**I**) Representative images of SA-β-galactosidase staining of MDFs in each group. (**J**) Cellular metabolic activity was measured using the 3-(4,5-dimethylthiazol-2-yl)-2,5-diphenyltetrazolium bromide assay. (**K**–**Q**) Western blotting representative images and bar charts showing the total proteins of MMP9, MMP3, P53, P16, P21, and γ-H2X of MDFs in each group. (**R**–**T**) Western blotting representative images and bar charts showing the total proteins of Collagen-I and III of MDFs in each group. Data are presented as mean ± SEM. *n* = 5 independent experiments. # denotes *p <* 0.05 vs. the control group; & indicates *p <* 0.05 vs. the UVB group.

The inhibition of GSK-3β expression using GSK-3β siRNA further revealed the canonical Wnt signaling contributes to ISE’s anti-photoaging properties. Knockdown of GSK-3β by siRNA significantly decreased its mRNA and protein expression, while elevating activated β-catenin and Wnt2 protein levels in fibroblasts (Figure 4A–C). In contrast, treatment with ISE or GSK-3β siRNA alone markedly promoted the expression of activated β-catenin and Wnt2, whereas their combined application did not produce an additive inhibitory effect (Figure 4D–G).

Compared with the events in the control group, UVB irradiation significantly increased the β-galactosidase-positive area and markedly decreased cell viability. GSK-3β inhibition or treatment with ISE alone reduced β-galactosidase positivity and improved cell viability. However, combined treatment with ISE and GSK-3β siRNA did not enhance the protective effects of ISE compared with the effects of ISE treatment alone (Figure 4H–J). Western blotting confirmed that, compared with ISE-alone treatment, combining ISE with GSK-3β inhibition did not produce additional reductions in UVB-induced SASP secretion (MMP3 and MMP9, Figure 4K–M), did not further attenuate activation of the DNA damage-related P53-P21 axis (Figure 4K,N–Q), nor did it further restore Collagen I and III levels (Figure 4R–T).

The results from Figure 3 and Figure 4 indicate that ISE exerts anti-photoaging effects through downregulation of GSK-3β, leading to enhanced activation of the Wnt/β-catenin pathway.

### 2.5. The Protective Effect of ISE Against UVB-Induced Mitochondrial Dysfunction Depended on Wnt/β-Catenin Signaling

**Figure 5:**
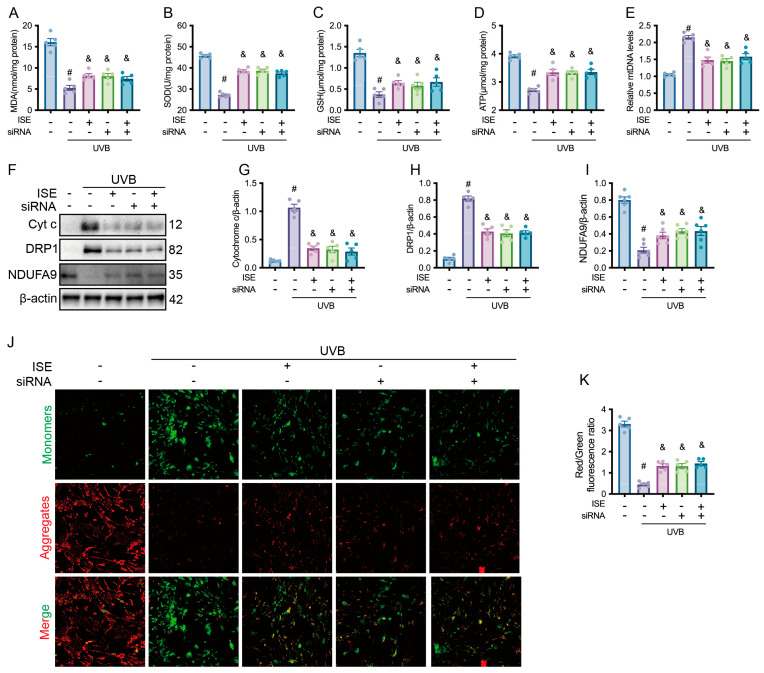
Effect of siRNA targeting β-catenin on ise in preventing UVB-induced mitochondrial dysfunction in vitro. (**A**–**E**) Bar charts showing malondialdehyde, superoxide dismutase, glutathione, ATP, and mtDNA levels in MDFs in each group. (**F**–**I**) Representative Western blotting and quantitative bar graphs showing Cytochrome c, DRP1, and NDUFA9 proteins in MDFs among different groups. (**J**,**K**) Representative images of JC-1 staining and quantification of the red/green fluorescence intensity ratio. Scale bars = 50 µm. Aggregates, red; Monomers, green. Data are presented as mean ± SEM. *n* = 5 independent experiments. # denotes *p <* 0.05 vs. the control group; & indicates *p <* 0.05 vs. the UVB group.

Furthermore, we identified that Wnt/β-catenin signaling mediates the protective effects of ISE against UVB-induced mitochondrial dysfunction. UVB irradiation markedly lowered cellular antioxidant capacity, as reflected by reduced MDA, SOD, GSH, and ATP levels. Notably, either ISE treatment alone or GSK-3β inhibition significantly restored these antioxidant levels and ATP compared with the UVB group. However, combined treatment with ISE and GSK-3β inhibition did not cause these increases relative to those in the ISE treatment alone (Figure 5A–D). The mtDNA analysis showed that the combined treatment with ISE and GSK-3β siRNA (1.49 ± 0.20) did not differ significantly from the ISE-only group (1.58 ± 0.20, Figure 5E). Treatment with either ISE or GSK-3β siRNA alone alleviated UVB-induced increases in Cytochrome c and DRP1 and restored NDUFA9 expression. Notably, co-treatment with ISE and GSK-3β siRNA did not confer additional benefits (Figure 5F–I). JC-1 staining revealed that UVB exposure decreased mitochondrial aggregates (red fluorescence) and increased mitochondrial monomers (green fluorescence). Treatment with ISE alone markedly reduced monomers and restored aggregates. Conversely, combined treatment with ISE and GSK-3β inhibition did not enhance the regulatory effects of ISE on the aggregate/monomer ratio compared with the ISE-only group (Figure 5J,K).

### 2.6. ISE Reduced UVB-Triggered Skin Photoaging In Vivo

**Figure 6:**
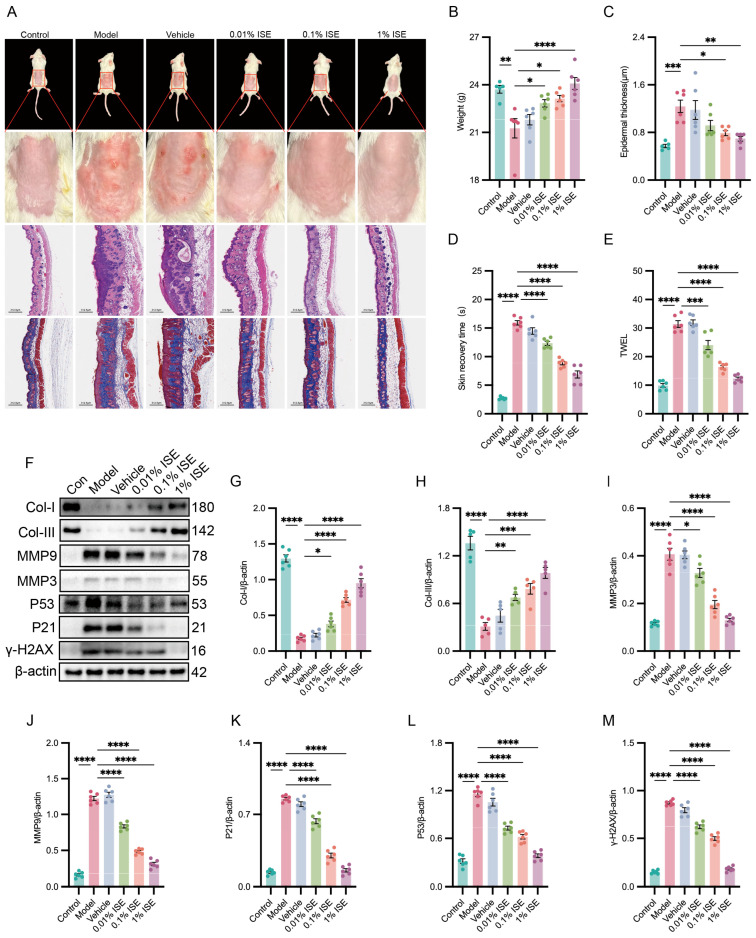
Assessment of the protective effect of ISE on UVB-induced skin photoaging in vivo. (**A**) Representative photographs of BALB/c mouse dorsal skin after 28 days of UVB irradiation, with hematoxylin and eosin- and Masson-stained sections shown for histological evaluation. (**B**) Data on the body weight of BALB/c mice after 28 consecutive days of irradiation. (**B**–**E**) Endpoint measurements included body weight, back skin thickness, recovery time, and transepidermal water loss in each group. (**F**–**M**) Representative Western blotting and quantitative bar graphs showing Collagen 1, Collagen 3, MMP9, MMP3, P53, P21, and γ-H2AX protein levels in the mouse dorsal skin among different groups. Data are presented as mean ± SEM. *n* = 6 independent experiments. * p < 0.05, ** *p <* 0.01, *** *p <* 0.001, **** *p <* 0.0001.

Mice received UVB irradiation on the dorsal skin every other day for 6 weeks and were subsequently treated with different concentrations of ISE. Using body weight as a systemic health indicator, we found that UVB irradiation significantly reduced body weight compared with control mice (21.27 ± 0.61 g vs. 23.72 ± 0.24 g) (Figure 6B). This reduction was dose-dependently attenuated by ISE treatment at 0.01% (22.83 ± 0.23 g), 0.1% (23.13 ± 0.20 g), and 1% (24.08 ± 0.37 g). Macroscopic observation revealed that ISE markedly alleviated UVB-induced skin dryness and erythema (Figure 6A). Consistently, epidermal thickness analysis (Figure 6C) and H&E staining (Figure 6A) demonstrated that 0.1% and 1% ISE significantly suppressed UVB-induced epidermal hyperplasia. Masson staining further revealed that UVB irradiation caused marked damage to dermal elastic fibers, characterized by fragmentation, abnormal proliferation, and disorganized architecture, whereas these alterations were markedly improved by 0.1% and 1% ISE treatments (Figure 6A). The assessment of skin barrier function revealed that UVB irradiation significantly prolonged skin recovery time and increased transepidermal water loss compared with controls. ISE treatment significantly reduced both parameters in a dose-dependent manner (Figure 6D,E), indicating effective protection against UVB-induced skin barrier dysfunction. Consistent with the epidermal thickness analysis and H&E staining, Western blotting revealed that Collagen I and III levels in mouse skin tissues were reduced by UVB exposure, whereas ISE treatment at various concentrations significantly restored their expression (Figure 6F–H). In addition, UVB exposure significantly increased SASP-related (MMP3 and MMP9) and senescence-associated (P53, P21, and γ-H2AX) markers in skin tissues, which were dose-dependently ameliorated by ISE (Figure 6F,I–M).

## 3. Discussion

Mitochondrial dysfunction and the resulting DNA damage are central events in photoaging, driving the excessive secretion of senescence-associated inflammatory factors [10]. These mediators amplify chronic cutaneous inflammation, promote extracellular matrix degradation, and disrupt skin architecture and function, leading to wrinkle formation, dermal thinning, and loss of skin elasticity [8,20]. Although mitochondrial dysfunction is critically involved, therapeutic strategies aimed at this process remain difficult to implement in the context of photoaging. Our results demonstrate that ISE confers protection against photoaging by modulating mitochondrial dysfunction. Moreover, restoration of UVB-suppressed Wnt/β-catenin signaling plays a pivotal mechanistic role in the anti-photoaging and mitochondrial-protective actions of ISE. Mechanistically, ISE relieves suppression of β-catenin through downregulation of GSK-3β.

Our data demonstrate that topical ISE (0.01–1%) dose-dependently alleviated erythema, wrinkling, and tissue damage in UVB-induced photoaged mice. In photoaged skin, wrinkle formation and erythema result from impaired collagen synthesis in senescent fibroblasts and sustained secretion of SASP factors, the latter drive collagen and elastin degradation [8]. Consistent with this mechanism, ISE markedly attenuated UVB-induced fibroblast senescence. In vitro, UVB exposure markedly increased SA-β-gal–positive fibroblasts and SASP markers (MMP3 and MMP9), whereas these effects were effectively attenuated by ISE treatment. Notably, the aforementioned phenotypic improvements were achieved by administering ISE exclusively post-irradiation in both the in vitro and in vivo models. Consequently, the photoprotective efficacy of ISE cannot be attributed to a primary sunscreen effect (i.e., functioning as a direct UV absorber during exposure).

Mitochondria are essential for cellular energy metabolism, and their functional integrity depends on an efficiently regulated intracellular antioxidant system [21]. During photoaging, excessive oxidative stress disrupts mitochondrial redox homeostasis, causing mitochondrial membrane potential imbalance, impaired collagen synthesis, and enhanced secretion of aging-associated phenotypic factors that accelerate collagen degradation [21,22]. Although targeting mitochondrial oxidative stress mitigates skin photoaging, effective therapeutic agents remain limited, highlighting the need for novel strategies targeting mitochondrial dysfunction. ISE suppresses neutrophil extracellular trap formation by reducing cellular ROS levels [18]; however, its role in other mitochondrial dysfunction-related conditions remains unexplored. In the present study, ISE dose-dependently restored antioxidant capacity in UVB-induced photoaged cells, enhancing the production of SOD, GSH, and MDA (Figure 2A–C).

During photoaging, UVB energy or UVB-induced excessive oxidative stress causes extensive DNA damage [4,5]. DNA double-strand breaks promptly activate H2AX phosphorylation, leading to elevated γ-H2AX levels that reflect early activation of DNA damage signaling [6,23]. The subsequent activation of the ATM/ATR signaling cascade stabilizes and activates p53, which transcriptionally upregulates the cyclin-dependent kinase inhibitor p21 [24,25]. Accordingly, DNA damage severity can be evaluated by assessing the activation of the γ-H2AX–p53–p21 axis. Our findings indicate that ISE exerts a protective effect against DNA damage, as evidenced by the downregulated γ-H2AX–p53–p21 axis in the MDF cells treated with ISE after UVB irradiation compared with that in the irradiated cells without ISE treatment (Figure 1C–E). Notably, extended activation of DNA damage pathways results in p53-driven mitochondrial dysfunction, leading to impaired electron transport and increased ROS leakage [26,27]. In parallel, the continuous utilization of NAD^+^ and antioxidant molecules during DNA repair, together with mitochondrial DNA damage, weakens cellular redox homeostasis, establishing a self-amplifying loop between DNA damage and oxidative stress [28,29]. Here, we demonstrated that ISE substantially alleviated UVB-induced mitochondrial dysfunction through restoring NDUFA9 (a critical subunit of the mitochondrial oxidative phosphorylation complex), suppressing DRP1 expression (a major regulator of mitochondrial fission) and reducing Cytochrome c, suggesting a protective role against photoaging partially through restoring mitochondrial function (Figure 2F–J). The unique cyclodepsipeptide structure of ISE suggests the presence of previously unrecognized mechanisms governing mitochondrial regulation, along with additional pharmacological activities relevant to photoaging.

Wnt signaling is widely recognized for its fundamental involvement in a broad range of cellular functions and its association with multiple human disorders [30]. Increasing evidence underscores its capacity to promote health and delay aging, with therapeutic modulation of Wnt signaling pathway showing therapeutic potential in age-associated diseases, including Alzheimer’s disease and osteoporosis [31]. In the skin, the activation of the Wnt/β-catenin pathway protects against UVB-induced photoaging and facilitates cutaneous wound healing [32]. The present study revealed that ISE upregulated Wnt/β-catenin signaling, suggesting this pathway underlies its regulatory effects on photoaging. GSK-3β acts as a central inhibitory modulator of the classical Wnt/β-catenin pathway. Basally, GSK-3β phosphorylates β-catenin within the destruction complex, leading to its ubiquitination and subsequent proteasomal degradation [11]. The involvement of GSK-3β in controlling cellular senescence has been well documented. The persistent activation of GSK-3β suppresses Wnt/β-catenin signaling, enhances oxidative stress, and promotes mitochondrial dysfunction, facilitating p53/p21-dependent cell-cycle arrest and senescence [33,34]. In contrast, GSK-3β inhibition attenuates DNA damage responses, restores mitochondrial homeostasis, reduces ROS accumulation, and reactivates Wnt/β-catenin signaling [34,35]. These effects contribute to the alleviation of UV-induced photoaging and the preservation of fibroblast function, highlighting GSK-3β as a potential anti-aging therapeutic target. In this study, we suppressed GSK-3β expression using siRNA to verify that GSK-3β is a binding target of ISE and that the subsequent activation of Wnt/β-catenin signaling pathway represents a potential mechanism underlying its protective effects against photoaging and mitochondrial dysfunction (Figure 4 and Figure 5). GSK-3β has many other targets in fibroblasts; thus we cannot exclude the possibility that GSK-3β influences other pathways, including PI3K/Akt and NF-κB-dependent regulation of apoptosis [36] and skin inflammation [37], which may likewise contribute to its anti-aging effects in the skin. Our RNA-seq data and immunoblotting confirmed that Wnt/β-catenin pathway is an essential signaling mechanism for ISE’s anti-photoaging property; however, the detailed mechanism requires further investigation. The validation should include the use of adenoviral vectors or specific agonists to evaluate the significance of the PI3K/Akt and NF-κB pathways in mediating ISE’s anti-photoaging effects.

Our study demonstrates that ISE, a newly identified marine-derived compound, counteracts UVB-induced mitochondrial impairment and photoaging through suppression of GSK-3β and subsequent activation of Wnt/β-catenin signaling.

## 4. Materials and Methods

### 4.1. Chemical Structure of ISE

Isaridin E (ISE), a colorless crystalline compound with the molecular formula C_35_H_54_O_7_N_5_ (The chemical structure of ISE is depicted in Figure 1A) on the basis of the (+)-HR-ESIMS at *m*/*z* 656.4014 [M + H]^+^ (Figure S1A), was utilized in this study. The UV spectrum (Figure S1B) showed absorption at 198 nm. The ^1^H NMR spectrum (Figure S1C) displayed the signals for two amide protons (*δ*_H_ 7.28, 8.15), two *N*-methyl groups (*δ*_H_ 2.97, 3.15), and five α-amino protons (*δ*_H_ 4.10, 4.30, 4.70, 5.12, 5.35). The signals observed in the ^13^C NMR spectrum (Figure S1D) were assigned to six carbonyls (*δ*_C_ 168.8−174.2), five α-amino acid carbons (*δ*_C_ 53.9, 57.7, 61.1, 66.6, 73.3), and a monosubstituted aromatic ring carbon (*δ*_C_ 127.4−136.5), indicating the six fragments including 2-hydroxy-4-methylpentanoic acid (HMPA), proline (Pro), phenylalanine (Phe), NMe-valine (NMe-Val), NMe-valine (NMe-Val), and β-alanine (β-Ala). These information were agreed with the chemical structure of isaridin E which was a cyclic hexadepsipeptide. High-Performance Liquid Chromatography (HPLC) analysis confirmed the purity of ISE (Rt = 10.88 min) to be >98.5% (Figure S1E and Table S1).

### 4.2. Preparations of ISE

ISE was dissolved in dimethyl sulfoxide (DMSO) for in vitro experiments and stored at 4 °C, ensuring that the final DMSO concentration did not exceed 0.1%. For animal experiments, ISE was administered in a saline-based vehicle containing 10% Tween 80 and 15% propylene glycol, used as the control formulation. The concentrations tested were 0.01%, 0.1% and 1%. Various concentrations of ISE were topically and uniformly administered to the mice’s irradiated dorsal skin following UVB exposure.

### 4.3. Animals

Animal experiments were conducted in accordance with protocols approved by the Institutional Animal Care and Use Committee of Sun Yat-sen University (No. SYSU-IACUC-2025-001583). Five-week-old female BALB/c mice were obtained from GemPharmatech (Nanjing, China) and maintained at 22 ± 2 °C with 50 ± 10% humidity under a 12-h light/dark cycle.

### 4.4. UVB Irradiation and ISE Treatment

After one week of acclimatization, mice were divided into six groups (five animals per group): untreated control, UVB-exposed, UVB plus vehicle, and UVB combined with 0.01%, 0.1%, or 1% ISE. UVB exposure was performed three times per week using a 311-nm broadband lamp (PL-S9W/01, Philips, Amsterdam, The Netherlands) with stepwise dose escalation (36, 54, 72, and 108 mJ/cm^2^ over weeks 1, 2–4, 5–7, and 8–10, respectively). The vehicle or ISE formulations (200 µL) were topically applied to the dorsal skin once daily after UVB irradiation. No treatment was administered to control animals. The baseline concentration of 0.01% ISE was extrapolated from our in vitro cellular data. Furthermore, 0.1% and 1% formulations were concurrently established to compensate for the limited percutaneous absorption imposed by the skin barrier.

### 4.5. Histological Analaysis

Harvested skin samples were fixed in 4% paraformaldehyde for 48 h, sectioned, and subjected to Masson’s trichrome and hematoxylin–eosin staining for collagen visualization and pathological assessment, respectively. Histological images were obtained with a Pannoramic MIDI scanner (3DHISTECH, Budapest, Hungary).

### 4.6. Isolation and Culture of Mouse Dermal Fibroblasts

MDFs were obtained from mouse dorsal skin via enzymatic digestion using dispase and collagenase II and maintained in DMEM containing 10% fetal bovine serum and antibiotics under standard culture conditions (37 °C, 5% CO_2_). Passages 2–4 were selected for downstream assays.

### 4.7. UVB-Induced Cellular Senescence Model

Cells were washed twice with PBS and exposed to UVB irradiation (311 nm; PL-S9W/01, Philips, Amsterdam, The Netherlands) at a dose of 60 J/cm^2^ to induce cellular senescence. After irradiation, the PBS was removed and replaced with fresh culture medium containing different concentrations of ISE. The cells were then co-incubated with ISE for 24 h before subsequent analyses. Non-irradiated control cells underwent the same procedure except for UVB exposure and received fresh medium only. The concentration range of ISE was selected based on our previous study [17].

### 4.8. Detection of MDA, SOD and GSH

MDA (Servicebio, G4300, Wuhan, China), SOD (Biosharp, BL1748B, Beijing, China), and GSH (Servicebio, G4303, China) contents were quantified with assay kits

### 4.9. 3-(4,5-Dimethylthiazol-2-YL)-2,5-Diphenyltetrazolium Bromide (MTT) Assay

According to the manufacturer’s instructions (MCE, HY-15924, San Rafael, CA, USA), briefly, the medium was replaced with MTT solution (0.5 mg/mL), and cells were incubated at 37 °C for 4 h. After discarding the supernatant, formazan crystals were dissolved in DMSO. Absorbance was measured at 570 nm using a microplate reader, with viability expressed as a percentage of the non-irradiated control.

### 4.10. Synthesis of GSK-3β-siRNA

GSK-3β-specific siRNA and a negative control siRNA were obtained from Shanghai GenePharma (Shanghai, China). The interference (siRNA) sequences are listed in the Table 1 below.

### 4.11. RT-PCR Assay

Total RNA was extracted using TRIzol reagent (ThermoFisher, 15596026CN, Waltham, MA, USA), and 1 µg of RNA was reverse-transcribed for subsequent RT-PCR analysis. Conventional RT-PCR was performed with the AccessQuick RT-PCR System. GSK-3β was used to assess siRNA efficiency. ND1 was used to assess mitochondrial DNA content. β-actin serving as an internal control. Primer sequences were as follows (Table 2):

### 4.12. ATP Production Assay

Cellular ATP levels were quantified by firefly luciferase-based assay kit (Elabscience, E-BC-K157-M, Wuhan, China), and ATP concentrations calculated and normalized to total protein content using a standard curve.

### 4.13. JC-1 Staining

According to the manufacturer’s instructions: After incubation with JC-1 dye for 30 min at 37 °C, cells were washed and imaged under an inverted fluorescence microscope (Leica, DMI8, Wetzlar, Germany). Red and green fluorescence intensities were quantified using ImageJ.

### 4.14. RNA-Seq

In this study, RNA-seq served as an exploratory screening tool to identify signaling pathways modulated by ISE under UVB-induced conditions. Transcriptomic sequencing was performed on UVB-treated MDFs (UVB group) and UVB + 25 µM ISE-treated MDFs. RNA-seq was conducted by Sanya IDigital Biotechnology Co., Ltd. (Sanya, China). Li-braries were prepared using the NEBNext Ultra RNA Library Prep Kit for Illumina platforms. Transcript abundance was quantified and normalized using Salmon, reads were aligned to the reference genome, and differential expression of protein-coding genes was determined by comparing normalized expression levels between groups. To ensure rigorous mechanistic validation, crucial signaling pathways identified via this screening approach were subsequently confirmed using independent molecular assays that strictly incorporated a non-irradiated baseline control.

### 4.15. Western Blot

Cells or skin tissues were lysed in RIPA buffer (supplemented with protease and phosphatase inhibitors). Protein levels were determined through a BCA assay (ThermoFisher, 23225, USA). Proteins in equal quantities were separated by SDS–PAGE (Biosharp, BL565B, China) and then transferred to PVDF membranes (Merck Millipore, ISEQ00010, Darmstadt, Germany). Membranes were treated with 5–10% skim milk (Servicebio, GC310001, Wuhan, China) for 2 h at room temperature and then incubated overnight at 4 °C with primary antibodies as listed in Table 3.

The membranes were then incubated with the following secondary an-tibodies for 1 h at room temperature: goat anti-mouse IgG H&L (HRP) (1:6000, Servicebio, GB23301, China) or goat anti-rabbit IgG H&L (HRP) (1:6000, Servicebio, GB23303, China). Protein bands were detected using enhanced chemiluminescence (Merck Millipore, WBKLS0500, Germany) and imaged with a Bio-Rad imaging system. Band intensities were quantified using ImageJ (2.1.0, Madison, WI, USA), normalized to β-actin, and expressed as fold changes relative to the control.

### 4.16. Statistical Analysis

Data are presented as mean ± SEM. Group comparisons were performed using one-way ANOVA followed by the LSD post hoc test. *p* value of less than 0.05. was considered statistically significant.

## 5. Conclusions

This study provides evidence that Isaridin E, a novel marine-derived compound, attenuates UVB-induced fibroblast dysfunction and mitochondrial impairment, potentially through modulation of the GSK-3β/Wnt/β-catenin signaling pathway. Therefore, Isaridin E can be considered as a promising candidate compound for the prevention or treatment of skin photoaging.

## Figures and Tables

**Table 1:** Sequences of interference strands.

Group	Strand Type	Sequence
GSK-3β	Sense	5′-GGAGAGCCCAAUGUUUCAUTT-3′
	Antisense	5′-AUGAAACAUUGGGCUCUCCTT-3′
Control	Sense	5′-GCGCCAGUGGUACUUAAUATT-3′
	Antisense	5′-UAUUAAGUACCACUGGCGCTT-3′

**Table 2:** Primer sequences for RT-PCR.

Group	Strand Type	Sequence
GSK-3β	forward	5′-AAAGCGGCTGTTAGTCACTGG-3′
	reverse	5′-GACTTGGGAGGTATCCACATCC-3′
ND1	forward	5′-CTATGAATCCCCTTACCAATACCTC-3′
	reverse	5′-AGGAGCCGCTTATTAGGAGGAC-3′
β-actin	forward	5′-GAAATCGTGCGTGACATTA-3′
	reverse	5′-ACTCATCGTACTCCTGCTTG-3′

**Table 3:** Sources and dilution ratios of primary antibodies.

Primary Antibody	Manufacturer	Catalog	Dilution Ratio
Anti-P53	ABclone (Woburn, MA, USA)	A25915	1:1000
Anti-P21	Servicebio	GB115313	1:500
Anti-P16	Abcam (Cambridge, UK)	ab211542	1:500
Anti-γ-H2AX	ThermoFisher	A700053	1:1000
Anti-Collagen III	Servicebio	GB111629	1:1000
Anti-Collagen I	Servicebio	GB11022	1:1000
Anti-MMP3	Proteintech (Rosemont, IL, USA)	66338-1-Ig	1:1000
Anti-MMP9	ABclone	A26079	1:1000
Anti-Cytochrome c	Proteintech	10993-1-AP	1:1000
Anti-NDUFA9	Proteintech	20312-1-AP	1:1000
Anti-DRP1	Proteintech	12957-1-AP	1:1000
Anti-non phospho (active) β-catenin	CST (Danvers, MA, USA)	4270S	1:1000
Anti-β-catenin	ABclone	A19657	1:1000
Anti-GSK-3β	Servicebio	GB11099	1:1000
Anti-Wnt2	Proteintech	27214-1-AP	1:1000
β-actin	Servicebio	GB15003	1:4000

## Data Availability

All data included in this study are available upon request by con-tact with the corresponding author.

## Supplementary Materials

The following supporting information can be downloaded at: https://www.mdpi.com/article/10.3390/md24030112/s1, Figure S1A. The Mass spectrometric identification spectrum of ISE; Figure S1B. The UV spectrum of ISE; Figure S1C. The ^1^H NMR (400 MHz) spectrum of ISE in CDCl_3_; Figure S1D. The ^13^C NMR (400 MHz) spectrum of ISE in CDCl_3_. Figure S1E. The HPLC of isaridin E; Table S1: The HPLC of isaridin E.

## Abbreviations

The following abbreviations are used in this manuscript:

ISE	Isaridin E
ROS	Reactive oxygen species
GSH	Glutathione
SOD	Superoxide dismutase
mtDNA	Mitochondrial DNA
necrosis factor-alpha	TNF-α
HPLC	High-Performance Liquid Chromatography
GO	Gene Ontology
