# Supplementary material for: Isaridin E Protects Against UVB-Induced Photoaging by Activating Wnt/β-Catenin Signaling Pathway and Alleviating Mitochondrial Dysfunction
Source: Mar Drugs. 2026 Mar 18;24(3):112. doi: 10.3390/md24030112 (PMC13028182; doi:10.3390/md24030112)
Supplement: Supplementary file 1 [file marinedrugs-24-00112-s001.zip › marinedrugs-4167553-supplementary.pdf]

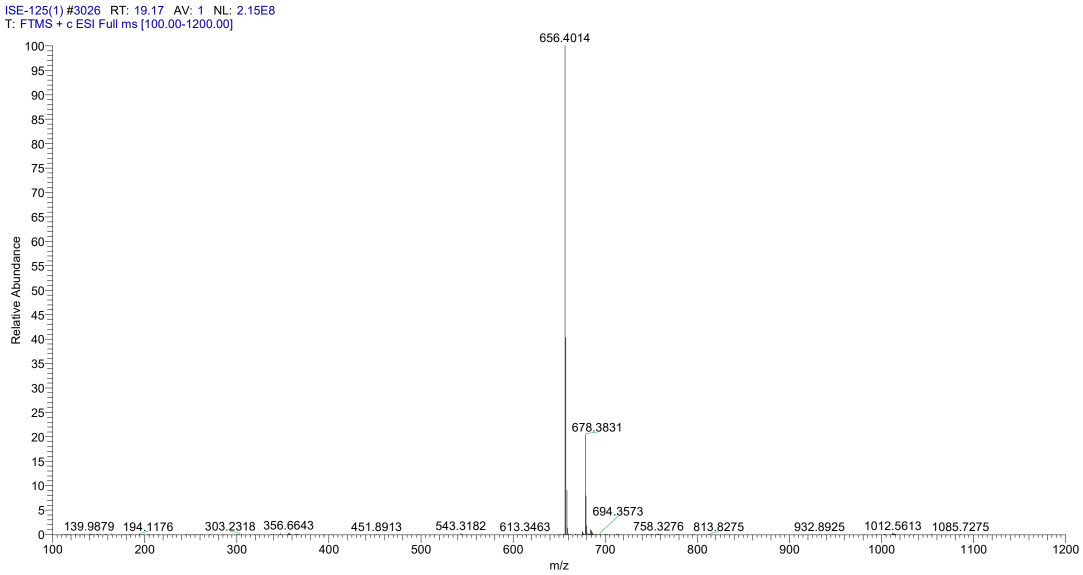

Figure S1A. The Mass spectrometric identification spectrum of ISE

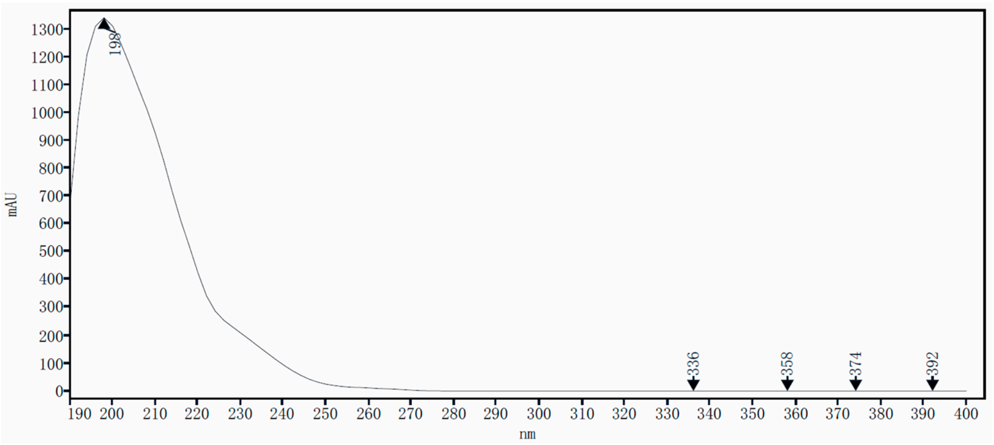

Figure S1B. The UV spectrum of ISE

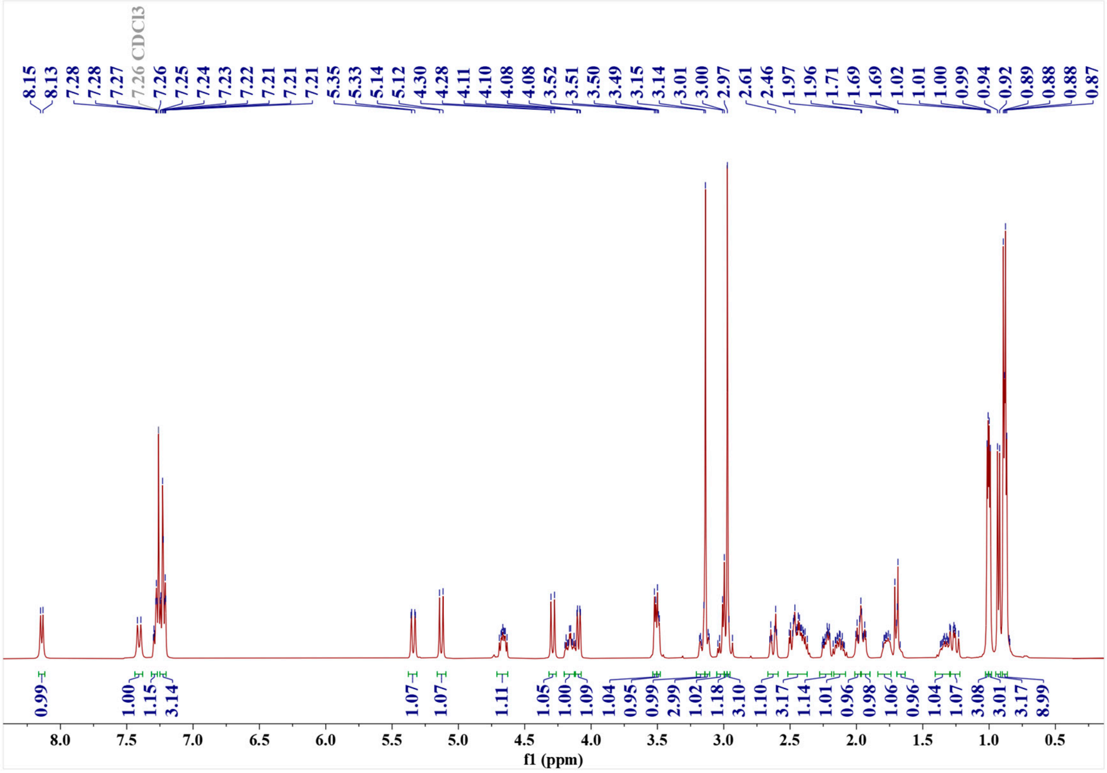

Figure S1C. The ^{1}H NMR (400 MHz) spectrum of ISE in $CDCl_3$.

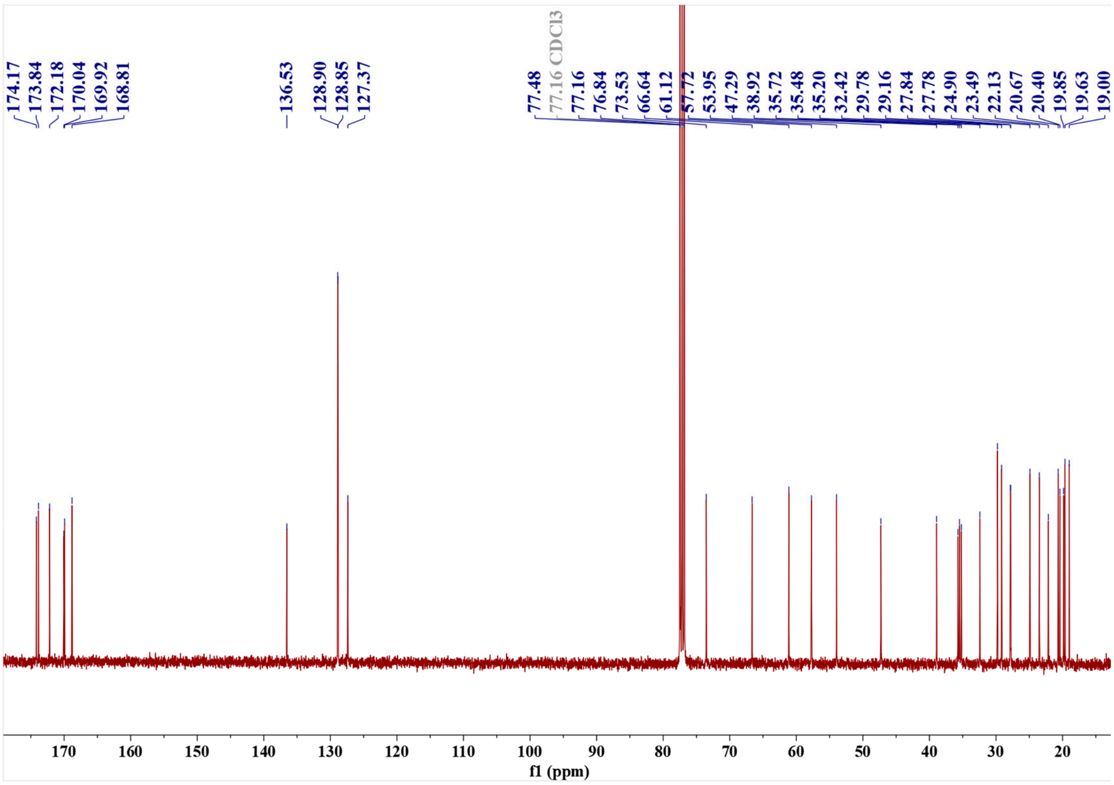

Figure S1D. The ^{13}C NMR (400 MHz) spectrum of ISE in $CDCl_3$.

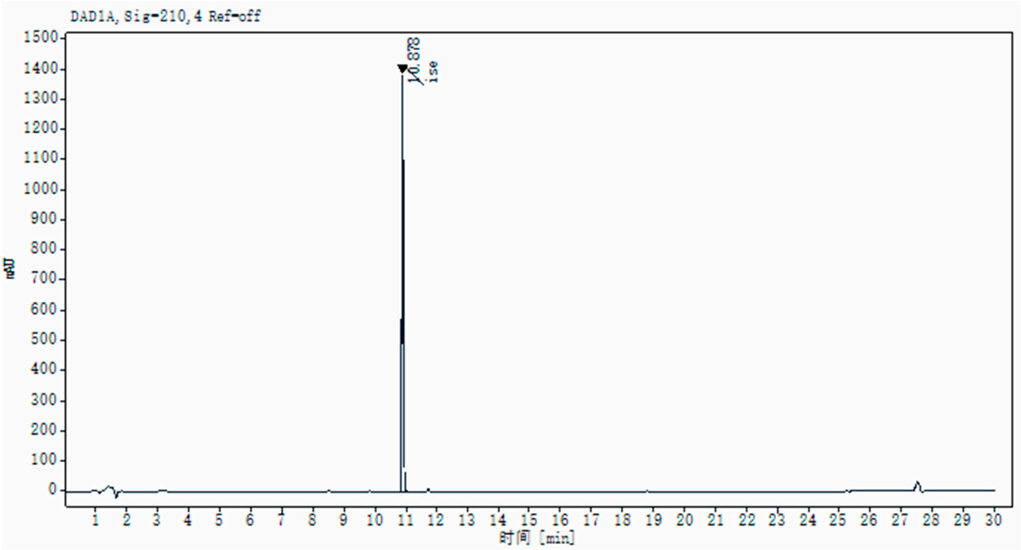

Columns: Agilent ZORBAX Eclipse Plus C18 (3.5 μm, 4.5×100 mm)；
Temperature: 30℃; Wavelength: 210nm; Flow rate: 0.8 mL/min; Volume: 10 μL.

Fig S1E. The HPLC of isaridin E

Table S1 The HPLC of isaridin E

Time	Aqueous phase A	Organic phase B acetonitrile
Starting conditions	70%	30%
2min	70%	30%
15min	0	100%
25min	0	100%
26min	70%	30%
30min	70%	30%
